# Association Analysis of Transcriptome and Targeted Metabolites Identifies Key Genes Involved in *Iris germanica* Anthocyanin Biosynthesis

**DOI:** 10.3390/ijms242216462

**Published:** 2023-11-17

**Authors:** Xiaojie Zhao, Yumeng Wu, Xiaoyu Zhang, Feng Tian, Fang Yu, Xue Li, Dazhuang Huang

**Affiliations:** Department of Landscape Architecture, Hebei Agricultural University, 2596 Lekai South Street, Baoding 071001, China; ylzxj@hebau.edu.cn (X.Z.); 18603225357@163.com (Y.W.); xixi20231010@163.com (X.Z.); 15630288081@163.com (F.T.); yufang202203@163.com (F.Y.); lx2468102023@163.com (X.L.)

**Keywords:** anthocyanins, bitone cultivar, flower coloration, structural genes, R2R3-MYB, association analysis

## Abstract

The anthocyanin biosynthetic pathway is the main pathway regulating floral coloration in *Iris germanica*, a well-known ornamental plant. We investigated the transcriptome profiles and targeted metabolites to elucidate the relationship between genes and metabolites in anthocyanin biosynthesis in the bitone flower cultivar ‘Clarence’, which has a deep blue outer perianth and nearly white inner perianth. In this study, delphinidin-, pelargonidin-, and cyanidin-based anthocyanins were detected in the flowers. The content of delphinidin-based anthocyanins increased with the development of the flower. At full bloom (stage 3), delphinidin-based anthocyanins accounted for most of the total anthocyanin metabolites, whereas the content of pelargonidin- and cyanidin-based anthocyanins was relatively low. Based on functional annotations, a number of novel genes in the anthocyanin pathway were identified, which included early biosynthetic genes *IgCHS*, *IgCHI,* and *IgF3H* and late biosynthetic genes *Ig F3′5′H*, *IgANS,* and *IgDFR*. The expression of key structural genes encoding enzymes, such as *IgF3H*, *Ig F3′5′H*, *IgANS,* and *IgDFR*, was significantly upregulated in the outer perianth compared to the inner perianth. In addition, most structural genes exhibited their highest expression at the half-color stage rather than at the full-bloom stage, which indicates that these genes function ahead of anthocyanins synthesis. Moreover, transcription factors (TFs) of plant R2R3-myeloblastosis (R2R3-MYB) related to the regulation of anthocyanin biosynthesis were identified. Among 56 R2R3-MYB genes, 2 members belonged to subgroup 4, with them regulating the expression of late biosynthetic genes in the anthocyanin biosynthetic pathway, and 4 members belonged to subgroup 7, with them regulating the expression of early biosynthetic genes in the anthocyanin biosynthetic pathway. Quantitative real-time PCR (qRT-PCR) analysis was used to validate the data of RNA sequencing (RNA-Seq). The relative expression profiles of most candidate genes were consistent with the FPKM of RNA-seq. This study identified the key structural genes encoding enzymes and TFs that affect anthocyanin biosynthesis, which provides a basis and reference for the regulation of plant anthocyanin biosynthesis in *I. germanica.*

## 1. Introduction

*Iris germanica* is the best-known and most horticulturally important species of iris. The color of its flowers is determined by the anthocyanin biosynthetic pathway and the carotenoid biosynthetic pathway. In cyanic iris flowers, blue, violet, and maroon hues are determined by delphinidin-type anthocyanins. Pelargonidin- and cyanidin-type anthocyanins are usually responsible for red and orange hues, but these pigments are deficient in *I. germanica* [1].

The anthocyanin biosynthetic pathway is well elucidated and conserved among seed plants. Anthocyanidins with diverse structures due to alternative substituents on the carbon positions of the two benzene rings are commonly grouped into six categories: malvidin, petunidin, pelargonidin, peonidin, delphinidin, and cyanidin [2]. Delphinidin, pelargonidin, and cyanidin are the three major anthocyanidins; petunidin and malvidin derive from delphinidin; and peonidin derives from cyanidin [3].

However, the different types of anthocyanins and their regulatory mechanisms vary by plant species. In flowers of *Iris ensata*, the major anthocyanins are malvidin and petunidin 3-(*p*-coumaroyl) rhamnosylglucoside-5-glucosides and non-acylated glucosides of these anthocyanidins [4]. In the 45 individuals of the beardless wild iris species *Iris domestica*, 29 anthocyanins have been identified, of which 10 anthocyanins derived from pelargonidin were first reported in iris species. In the petals of violet and purple groups, two to five major anthocyanins have been identified [5]. Cyanidin-, peonidin-, and pelargonidin-based anthocyanins are the major metabolites in Queen Rose [6]. The main pigment in the red petal phenotype of *Camellia japonica* is cyanidin-3-O-(6″-O-malonyl) glucoside, and cyanidin-3-O-rutinoside, peonidin-3-O-glucoside, cyanidin-3-O-glucoside, and pelargonidin-3-O-glucoside contribute to its deep coloration [7]. In *lycoris*, the major anthocyanin aglycone is pelargonidin-3-O-glucoside-5-O-arabinoside in the red petals [8].

The different types of anthocyanins are determined by the anthocyanin biosynthetic pathway, an extension of the general flavonoid pathway that is regulated by many structural genes. This pathway starts with chalcone synthase (CHS), the first key regulatory enzyme, which catalyzes the synthesis of naringenin chalcone. The subsequent enzymes are chalcone isomerase, flavanone 3-hydroxylase (F3H), flavonoid 3′-hydroxylase (F3′H), flavonoid 3′5′-hydroxylase (F3′5′H), dihydroflavonol 4-reductase (DFR), anthocyanidin reductase (ANS), and UDP-glucose: flavonoid 3-O-glucosyltransferase and others [9,10]. After biosynthesis, a series of transporters, including glutathione S-transferases [11,12], multidrug resistance-associated protein [13], and multidrug and toxic compound extrusion [14,15], transport anthocyanins into vacuoles to prevent their oxidation and to enable them to function properly as pigments.

In contrast to the well-conserved main pathway of flavonoid biosynthesis, the modification of anthocyanidins is family- or species-dependent and quite diverse. F3′H and F3′5′H are important branch point enzymes determining the B-ring hydroxylation of anthocyanins and consequently their diversification. The degree of B-ring hydroxylation is responsible for the hue of the anthocyanin pigment [4,16]. DFR catalyzes specific substrates and affects the composition and coloration of anthocyanin [10].

In most cases, the temporal and spatial control of anthocyanin biosynthesis is regulated by transcription factors (TFs). Myeloblastosis (MYB) is the most reported TF, which is crucial for mediating anthocyanin accumulation [17,18,19]. The subgroups of R2R3-MYB TFs were assumed to have conserved functions in the regulation of flavonoid metabolism. For instance, MYB subgroup 4 (SG4) was found to be able to inhibit the expression of late biosynthetic genes in the anthocyanin biosynthetic pathway [20]. Moreover, the ternary TF complex MBW formed by MYBs, basic helix–loop–helix (bHLH), and WD repeat (WDR) families can activate anthocyanin biosynthetic genes [21]. 

With a full understanding of the anthocyanin biosynthetic pathway, members of the flower and ornamental plant industry can create novel-colored varieties through genetic modification. For instance, transgenic blue/violet carnations, roses, and chrysanthemums have been developed by the transgene expression of a heterologous *F3′5′H* gene to produce B-ring-trihydroxylated anthocyanins [22,23,24]. However, limited knowledge of the genetic mechanism behind flower color in *I. germanica* inhibits such developments. 

**Figure 1:**
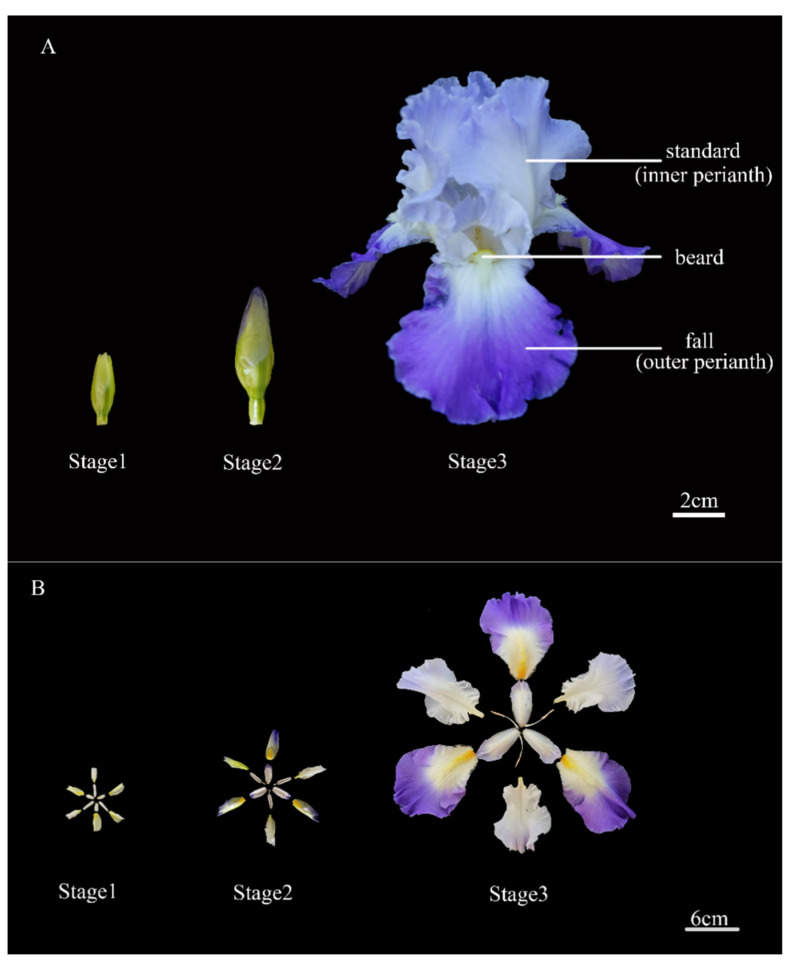
Morphological observation of *Iris germanica* cultivar ‘Clarence’ flowers during 3 developmental stages (standard, inner perianth; fall, outer perianth; stage 1, uncolored flower bud; stage 2, half-colored flower bud; and stage 3, full-bloom flower; F1, falls in stage 1; S1 standards in stage 1; F2, falls in stage 2; S2 standards in stage 2; F3, falls in stage 3; S3 standards in stage 3). (**A**) The phenotypes of the whole flower. (**B**) The phenotypes of the dissected flower.

The bitone cultivar Clarence, which has a blue outer perianth (falls) and nearly white inner perianth (standards) in the same flower (Figure 1), is an ideal cultivar in which to study the mechanism underlying anthocyanin synthesis in *I. germanica*. Here, we investigated profiles of anthocyanins and the transcriptome in the standards and falls at different developmental stages that contribute to the genetic modification of the anthocyanin biosynthetic pathway.

## 2. Results

### 2.1. Total Anthocyanin Content

**Figure 2:**
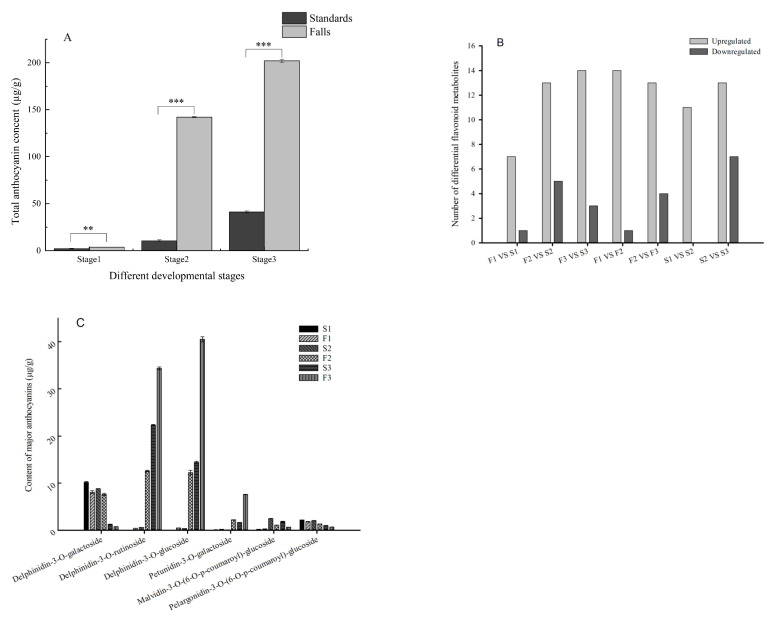
The number and content of anthocyanins in standards (inner perianths) and falls (outer perianths) during 3 developmental stages (stage 1, uncolored flower bud; stage 2, half-colored flower bud; and stage 3, full-bloom flower; F1, falls in stage 1; S1 standards in stage 1; F2, falls in stage 2; S2 standards in stage 2; F3, falls in stage 3; S3 standards in stage 3). (**A**) Contents of total anthocyanins among the comparison groups. ** *p* < 0.01, and *** *p* < 0.001. (**B**) Number of differential anthocyanins among the comparison groups. (**C**) Contents of major anthocyanins in standards and falls samples during 3 developmental stages. Each piece of data are the mean of three biological replicates.

The total anthocyanin content was detected at three developmental stages (stage 1, uncolored flower bud; stage 2, half-color flower bud; stage 3, full-bloom flower; Figure 1A,B). As the flower developed, the total anthocyanin content increased. Moreover, the total anthocyanin content was significantly higher in the falls than in the standards at all developmental stages (Figure 2A). This trend is consistent with the phenotype of a flower that has light standards and deeply colored falls. 

### 2.2. Identification and Quantification of Anthocyanin Components

A total of 31 anthocyanins were identified in *I. germanica* Clarence flowers, including flavonoids, delphinidins, cyanidins, pelargonidins, malvidins, peonidins, and petunidins (Table S1). The content of most anthocyanins was upregulated in the falls compared to the standards (Figure 2B). The predominant anthocyanins were delphinidin-3-O-glucoside, delphinidin-3-O-rutinoside, delphinidin-3-O-galactoside, petunidin-3-O-galactoside, malvidin-3-O-(6-O-p-coumaroyl)-glucoside, and pelargonidin-3-O-(6-O-p-coumaroyl)-glucoside (Figure 2C). 

Delphinidin-3-O-glucoside and delphinidin-3-O-rutinoside were initially present at low levels, but their content gradually increased until they peaked at stage 3. This trend was consistent with changes in the flower phenotype, which indicates that delphinidin-3-O-glucoside and delphinidin-3-O-rutinoside might play crucial roles in the formation of blue color. Delphinidin-3-O-galactoside accounted for 71.50% and 53.77% of total flavonoid metabolites in the standards and falls, respectively, at stage 1, but its content decreased over the course of development (Figure 2C). 

At stage 3, delphinidin-3-O-glucoside and delphinidin-3-O-rutinoside accounted for most of the total anthocyanin metabolites. The results show that in the standards, the content of delphinidin-3-O-rutinoside was higher than the content of delphinidin-3-O-glucoside in the falls, while in the falls, the content of delphinidin-3-O-rutinoside was lower than the content of delphinidin-3-O-glucoside (Figure 2C).

Pelargonidin- and cyanidin-based anthocyanins are usually responsible for red and orange hues [3]. In this study, pelargonidin-based anthocyanins were detected, but their content decreased as the flower developed. Of the pelargonidin-based anthocyanins, pelargonidin-3-O-(6-O-p-coumaroyl)-glucoside had the highest content, which was higher in the standards than in the falls (Figure 2C, Table S1). Three types of cyanidins were only detected at stage 2 or stage 3, and their content was quite low (Table S1). 

### 2.3. Overview of the Transcriptome Data

To determine gene expression, we performed RNA-seq analysis. A total of 126.8 Gb raw data and 410,713,043 clean reads were obtained through sequencing. In each library, the Q20 and Q30 values were greater than or equal to 97.2% and 92.3%, which indicates that the sequencing quality was sufficient for further analysis. Based on the high-quality reads, 157,702 unigenes were assembled.

### 2.4. DEGs in Standards and Falls at Different Developmental Stages and Gene Screening Using WGCNA

**Figure 3:**
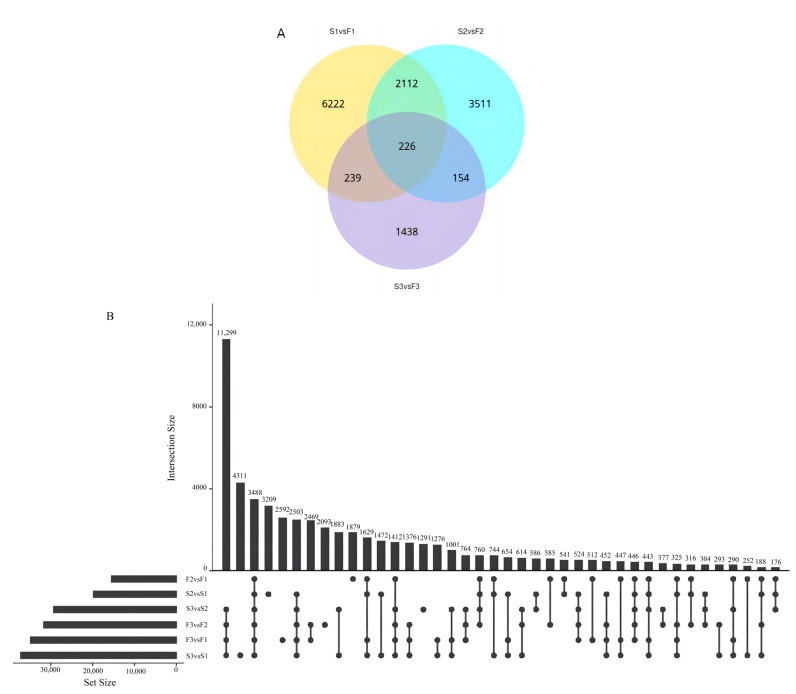
Analysis of differentially expressed genes (DEGs) in *Iris germanica.* (**A**) Venn diagram of DEGs identified at S1 vs. F1, S2 vs. F2, and S3 vs. F3. (**B**) Upset diagram of DEGs identified among 3 stages of falls and standards (F1, falls in stage 1; S1 standards in stage 1; F2, falls in stage 2; S2 standards in stage 2; F3, falls in stage 3; S3 standards in stage 3). Genes with |log2FoldChange| > 1 and *p* < 0.05 were considered to be differentially expressed.

To identify the DEGs involved in the coloration of iris flowers, we analyzed the FPKM values for each gene in the standards and falls at different developmental stages. A total of 6222, 3511, and 1438 DEGs were identified in pairwise comparisons of F1 vs. S1, F2 vs. S2, and F3 vs. S3, respectively (Figure 3A). The number of DEGs between the standards and falls was highest at developmental stage 1 and decreased as the flower developed. In the pairwise comparisons of the different developmental stages, the number of DEGs between stage 2 and stage 3 was higher than that between stage 1 and stage 2 for both the standards and falls (Figure 3B).

**Figure 4:**
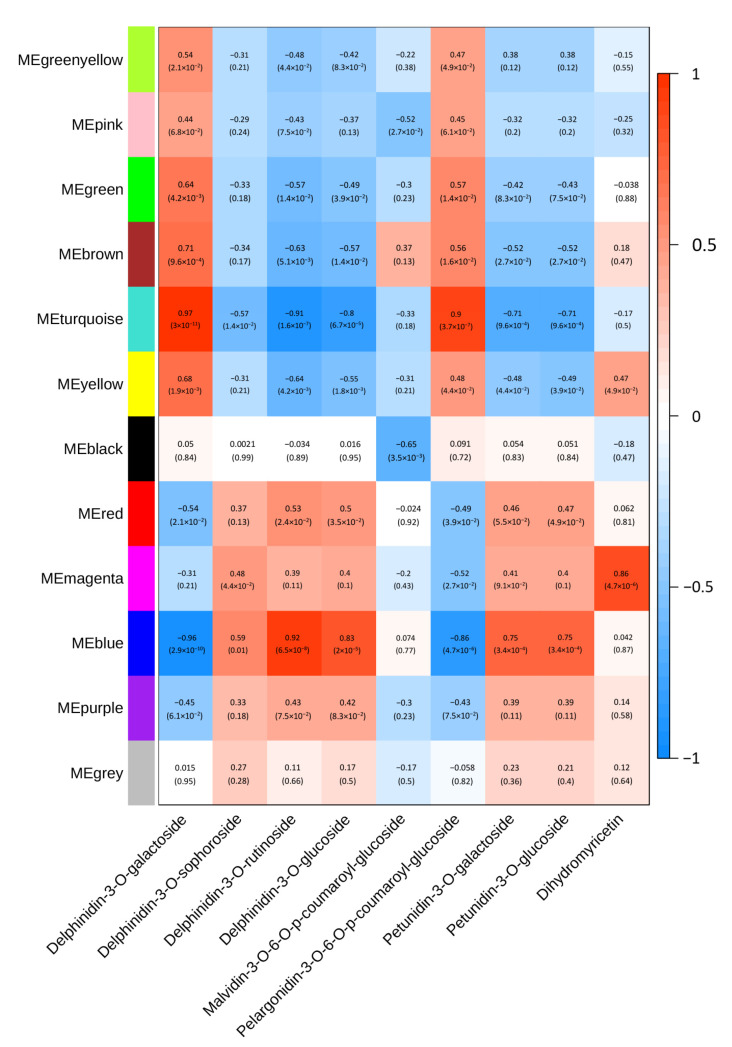
Module anthocyanin association analysis. Heatmap showing the correlation between modules and anthocyanins. The GS-value between a given module and anthocyanins is indicated by the color of the cell and the text inside the cell (the upper number is the value, and the lower number is the *p*-value). Red and blue indicate positive and negative correlations, respectively.

WGCNA was used to elucidate the gene expression in the falls and standards at different developmental stages. Based on the expression network analysis, a total of 12 modules were obtained. To screen out the key genes related to the anthocyanin biosynthetic pathway, we calculated associations between the modules and major anthocyanins; the correlation coefficients varied widely, from –0.91 to 0.97. The blue and turquoise modules had high PCCs with major anthocyanins. The blue module was positively correlated with delphinidin-3-O-glucoside (r^2^ = 0.83) and delphinidin-3-O-rutinoside (r^2^ = 0.92), which were prominent in the falls and standards at stage 3. In addition, the correlations between anthocyanins and the blue module were always opposite those for the turquoise module (Figure 4). In the KEGG analysis of the blue and turquoise modules, three terms, for example, KEGG: 00940 (phenylpropanoid biosynthesis), KEGG: 00941 (flavonoid biosynthesis), and KEGG: 00944 (flavone and flavonol biosynthesis), were identified, which indicates that genes in these modules play key roles in flavonoid or anthocyanin biosynthesis. With further analysis of the DEGs annotated to these three KEGG terms, the number of DEGs varied among the pairwise comparisons of F1 vs. S1, F2 vs. S2, and F3 vs. S3. Cluster-7660.80852, annotated as chalcone synthase, was identified in the blue modules with different expressions in both the pairwise comparisons of F1 vs. S1 and F2 vs. S2.

### 2.5. Correlations between DEGs and Anthocyanins

**Figure 5:**
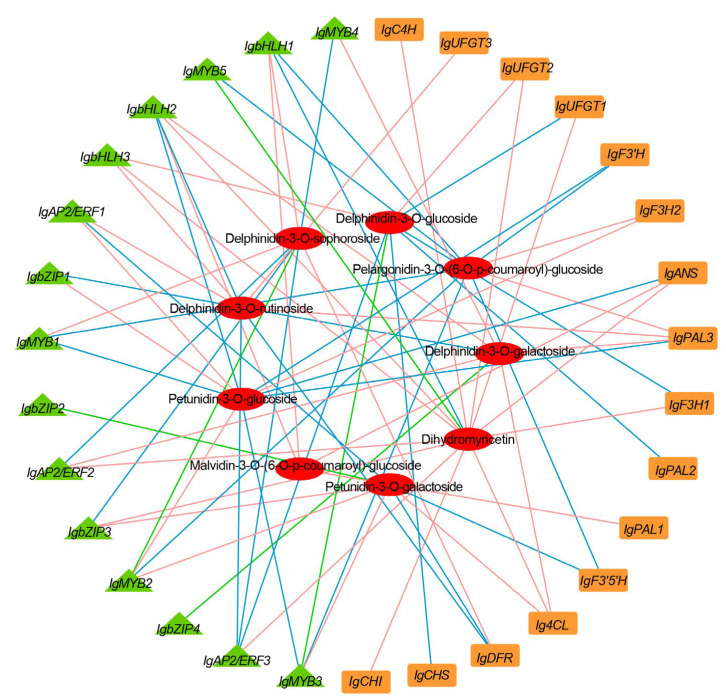
Correlation network of major metabolites and key structural genes and transcription factors involved in phenylpropanoid and flavonoid biosynthesis in *Iris germanica*. Pink lines represent a positive relation; blue lines represent a negative relation; and green lines show that the relation varied at different stages.

We performed a correlation analysis between the DEGs, encoding putative anthocyanin-modifying enzymes and transcriptome factors, and predominant anthocyanins to further understand their correlation. Predominant anthocyanins were positively or negatively correlated with the expression of several genes in the anthocyanin biosynthetic pathway. Delphinidin 3-*O*-glucoside and delphinidin-3-*O*-rutinoside, the main anthocyanins at full bloom, were positively related to *IgUFGT2* and *IgUFGT3*, respectively. Transcription factors (TFs) strongly related to key metabolites were also identified. Among these TFs, *IgbHLH3* correlated strongly with three predominant anthocyanins: delphinidin 3-*O*-glucoside, delphinidin-3-*O*-rutinoside, and dihydromyricetin (Figure 5).

### 2.6. Expression of Candidate Genes in the Anthocyanin Biosynthetic Pathway

**Figure 6:**
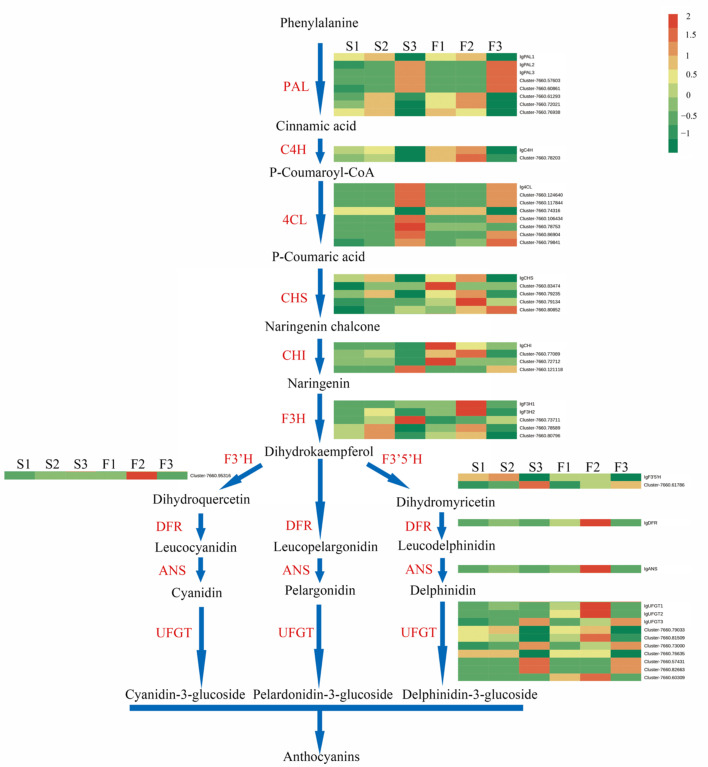
Structural genes regulated important enzymes identified in the anthocyanin biosynthesis pathway. PAL: phenylalanine ammonia lyase; C4H: cinnamate 4-hydroxylase; 4CL: 4-coumarate coenzyme A ligase; CHS: chalcone synthase; CHI: chalcone isomerase; F3H: flavonoid 3-hydroxylase; F3′H: flavonoid 3′-hydroxylase; F3′5′H: flavonoid 3′5′-hydroxylase; DFR: dihydroflavonol 4-reductase; ANS: anthocyanin synthase; FLS: flavonol synthase; LAR: leucoanthocyanidin reductase; UFGT: UDP-glucose: flavonoid-O-glycosyltransferase. Grids with a color scale from green to yellow to red represent the gene expression of the DEGs from low to medium to high. The sample names are provided above the heat map (F1, falls in stage 1; S1 standards in stage 1; F2, falls in stage 2; S2 standards in stage 2; F3, falls in stage 3; S3 standards in stage 3).

The expression of candidate genes involved in the anthocyanin biosynthetic pathways was analyzed. A total of 46 candidate genes were assigned to this pathway based on the annotation (Figure 6, Table S2). Several key structural genes in the anthocyanin biosynthetic pathway, including *F3H*, *DFR*, *ANS*, *anthocyanidin 3-O-glucosyltransferase*, and *anthocyanin 5-O-glucosyltransferase*, exhibited the highest expression in the falls at stage 2 (Figure 6), which indicates that these genes enhance the biosynthesis of anthocyanins in the falls at stage 2 (half-color) rather than at stage 3 (full bloom).

At stage 1 and stage 2, *IgF3H*, *IgANS*, *IgUFGT1*, and *IgUFGT2* were at least eight-fold less abundant in the standards than in the falls (Table S2). The expression of these genes was concurrent with the accumulation of the total anthocyanins and major delphinidin-based anthocyanins. Moreover, the expression of certain candidate genes encoding the same enzymes varied, which indicates that further verification is needed to screen out unrelated genes. 

### 2.7. Identification of MYB Transcription Factors Involved in Anthocyanin Biosynthesis

**Figure 7:**
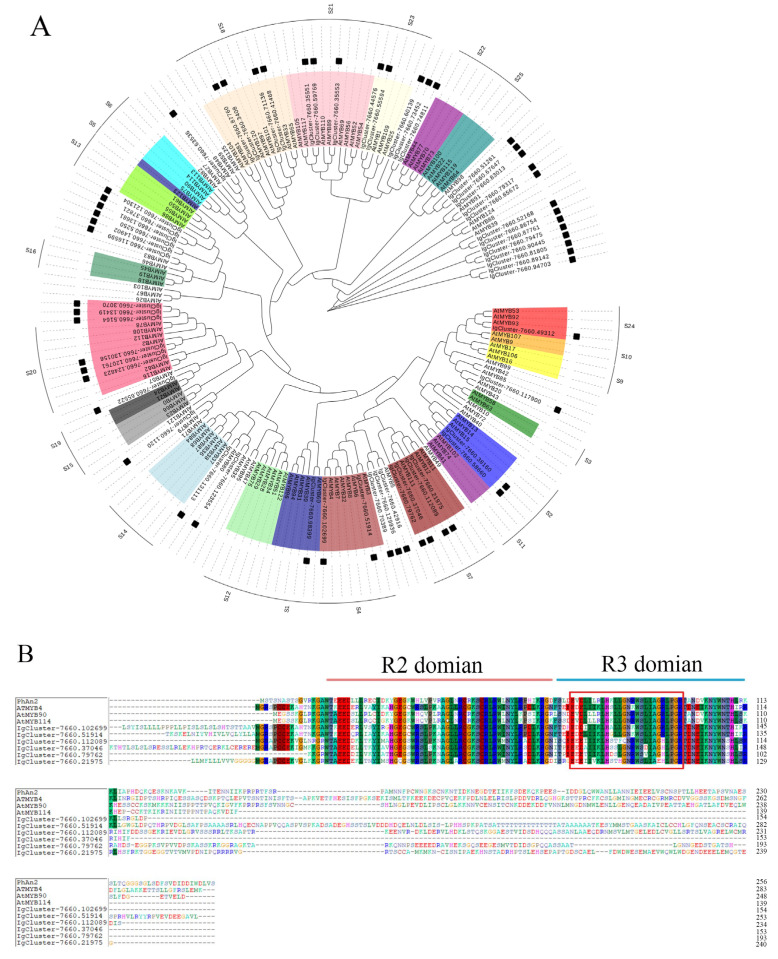
(**A**) Phylogenetic tree of putative R2R3-MYBs in *Iris germanica* and MYBs from *Arabidopsis thaliana*; the solid black squares represent the putative R2R3-MYBs in *Iris germanica*. (**B**) Amino acid sequence alignment of the R2R3-MYBs from *I*. germanica, *A*. thaliana, *Petunia* × *hybrida*. AtMYB4/90/114 (*Arbidopsis thaliana*, NP_195574, NP_176813, and NP_176812, respectively) and PhAn2 (*Petunia* × *hybrida*, AAF66727.1).

In this study, a total of 174 genes were identified based on information on functional annotations of RNA-seq data, among which, 56 R2R3-MYB genes were selected to construct a phylogenetic tree with R2R3-MYB genes from *Arabidopsis*. The 24 R2R3-MYB genes in *I. germanica* were clustered into function-annotated subgroups of *Arabidopsis*. Cluster-7660.102699 and Cluster-7660.51914 belonged to MYB subgroup 4 (SG4), which were able to inhibit the expression of late biosynthetic genes in the anthocyanin biosynthetic pathway, such as *DFR* and *ANS*. Cluster 7660.112089, Cluster-7660.79762, Cluster-7660.37046, and Cluster-7660.21975 belonged to subgroup 7 (SG7), with them positively regulating early biosynthetic genes in the anthocyanin biosynthetic pathway, such as *CHS*, *CHI*, and *F3H* [20]. The rest of the function-annotated MYBs were grouped into 21 other subgroups (Figure 7A). The alignments of the amino acid sequence revealed that putative R2R3-MYBs of *I. germanica* contained conserved R2 and R3 domains (Figure 7B).

### 2.8. Confirmation of Candidate DEGs through qRT-PCR Analysis

**Figure 8:**
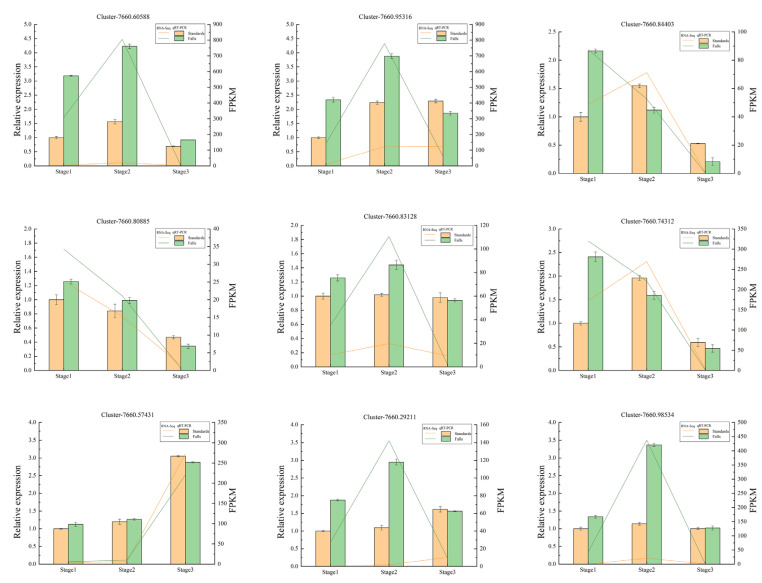
Quantitative real-time PCR (qRT-PCR) and fragments per kilobase per million (FPKM) of 9 DEGs identified by RNA sequencing (RNA-Seq). The *y*-axis on the left represents the relative gene expression levels (2^−∆∆*C*t^) analyzed by qRT-PCR; the *y*-axis on the right shows the FPKM value obtained by RNA-seq. The *x*-axis represents the sample of standards and falls in different stages.

The relative expression profiles of most candidate genes were consistent with the FPKM of RNA-seq. There are some discrepancies between qRT-PCR and RNA-seq at particular stages of a few genes. For instance, the expression of Cluster-7660.60588 and Cluster-7660.98534 in the standards was barely detected by qRT-PCR, but RNA-seq determined the expression of this gene (Figure 8).

## 3. Discussion

In this study, significant differences in the total content of anthocyanins between the standards and falls were found, which are responsible for color differences in the cultivar Clarence. The predominant anthocyanins identified in the flowers of the cultivar Clarence were delphinidin-based anthocyanins, such as delphinidin-3-O-glucoside, delphinidin-3-O-rutinoside, and delphinidin-3-O-galactoside. Pelargonidin-based anthocyanins were also detected, but their content was relatively low. Cyanidin-based anthocyanins were barely detected. Given the strict substrate specificity of DFR [10,25,26], we assume that DFR reduces dihydroflavonols primarily to delphinidin-based anthocyanins and less often to pelargonidin and cyanidin-based anthocyanins in Clarence. 

Pelargonidin- and cyanidin-based anthocyanins are usually responsible for red and orange hues [1]. A red-colored iris flower does not exist naturally because of a lack of cyanidin-based anthocyanins, which are usually predominant in magenta/red flowers [3]. The low content of pelargonidin- and cyanidin-based anthocyanins might explain why *I. germanica* lacks a natural ability to produce or accumulate certain pigments, which prevents it from producing red flowers. Genetic modification of the genes that regulate the synthesis of pelargonidin- and cyanidin-based anthocyanins could enable the production of red iris flowers.

At stage 3 (full bloom), delphinidin-3-O-glucoside and delphinidin-3-O-rutinoside accounted for most of the total anthocyanin metabolites, which indicates that these two anthocyanins contribute to the main pigment in flowers. It is interesting that in the standards, delphinidin-3-O-rutinoside was more prominent than delphinidin-3-O-glucoside. However, in the falls, delphinidin-3-O-glucoside and delphinidin-3-O-rutinoside showed the opposite trend (Figure 2C). In the anthocyanin biosynthesis reference pathway of KEGG, delphinidin-3-O-glucoside can transform into delphinidin-3-O-rutinoside under the catalyzing of anthocyanidin-3-glucoside rhamnosyltransferase. The study of *Lobelia erinus* revealed that due to a 5 bp nucleotide deletion of rhamnosyltransferase genes, rhamnosyltransferase cannot catalyze the rhamnosylation of delphinidin-3-O-glucoside, which was not further modified toward lobelinins; the cultivar Aqua Lavender showed a mauve flower [27]. The high accumulation of delphinidin-3-O-rutinoside in the standards might be related to the light coloration in the cultivar. 

In this study, we attempted to elucidate the role of key candidate genes in the anthocyanin biosynthetic pathway related to flower color. Key structural genes, such as *F3H*, *DFR*, and *ANS*, exhibited the highest expression at stage 2 (half-color) and were downregulated at stage 3 (full bloom). This is consistent with the expression of these genes in chili pepper, which also peaks prior to ripening and then is downregulated [28]. In the study of *Iris bulleyana*, the downregulation of anthocyanin 3-O-glucosyltransferase (3GT) and anthocyanin 5-O-glucosyltransferase (5GT) genes was assumed to hinder the accumulation of anthocyanins in white flower [29]. In our study, the expression of key structural genes encoding enzymes, such as *IgF3H*, *Ig F3′5′H*, *IgANS*, and *IgDFR*, was significantly upregulated in the blue falls compared to the white standards.

Given their overlapping expression with the anthocyanin content, *IgCHS*, *IgF3H*, *IgANS*, *IgUFGT1*, and *IgUFGT2* in the anthocyanin synthetic pathway play key roles in anthocyanin accumulation in Clarence. Of these genes, *CHS* is the primary rate-limiting enzyme in anthocyanin biosynthesis that mediates the synthesis of naringenin chalcone from 4-coumaroyl-CoA and malonyl-CoA [30,31]. The high expression of *CHS* provides sufficient amounts of precursor compounds for anthocyanin accumulation [6,32]. In contrast, silencing *CHS* reduces the content of anthocyanin and unpigmented fruit tissues in strawberry [33]. In the study of Han et al., *3GT* and all *DFR*, *Leucoanthocyanidin reductase* (*LAR*), and *anthocyanidin reductase* (*ANR*) genes showed high expression levels in *Iris pallida* compared with those in *I. germanica*, which might be responsible for flavonoids, especially flavonol and anthocyanin accumulation in *I. pallida* [34].

*F3H* is an early biosynthetic gene involved in the biosynthesis of pelargonidin-, cyanidin-, and delphinidin-based anthocyanins. In this study, the expression of *IgF3H* was 100-fold higher in the falls than in the standards at stage 1 and stage 2 (Table S2). This suggests that the different anthocyanin content between the standards and falls might be regulated by early biosynthetic genes. In *Camellia japonica*, a higher expression of *ANS* enhances anthocyanin accumulation [7]. To further verify the functions of SmANS when it was overexpressed in S. miltiorrhiza Bge f. alba, the purple-red phenotype was restored by increasing the anthocyanin concentration [35].

TF-regulated anthocyanin biosynthesis has been identified in many plant species [36,37,38]. The regulation of anthocyanin biosynthesis by MBW complexes, in which MYB together with bHLH and WD40 factors binds to the promoters of structural genes, is well documented [39,40,41]. In this study, of the identified TFs, *IgbHLH3* was strongly correlated with three predominant anthocyanins. This is consistent with previous research that shows that a high expression of *bHLHs* always co-occurs with an increasing expression of structural genes and anthocyanin content [42,43,44].

In this study, six R2R3-MYBs were grouped together with subgroup 4 and subgroup 7 of function-annotated R2R3-MYB genes from *Arabidopsis*. MYB Cluster-7660.102699 was grouped into subgroup 4, which was able to inhibit the expression of genes in the anthocyanin biosynthetic pathway [20,36]. The expression level of Cluster-7660.102699 at stage 3 was higher than that at stage 2 (Table S2), which showed a contrary trend with most identified structural genes, such as *IgF3H*, *IgANS,* and *IgDFR*, in the anthocyanin biosynthetic pathway. This result indicates that Cluster-7660.102699 might be a negative regulator of anthocyanin synthesis in *I. germanica*. In apple, with the assistance of ERA sequence, the overexpression of *MdMYB16* inhibited *MdUFGT* and *MdANS* expression and anthocyanin synthesis in red-fleshed callus [45].

## 4. Materials and Methods

*Iris germanica* ‘Clarence’ was planted in the nursery garden of Hebei Agricultural University in Baoding, China (38°49′30″ N, 115°26′44″ E), in 2022. Inner perianth (standards) and outer perianth (falls) of the same flower at three developmental stages (stage 1, uncolored flower bud; stage 2, half-color flower bud; stage 3, full-bloom flower; Figure 1) were collected to observe the phenotype and extract RNA and metabolites. The flowers were sampled and pooled from three individual plants, and three replicates were set for each stage from April to May 2022. All samples were immediately frozen in liquid nitrogen and then stored at –80 °C.

### 4.1. Measurement of Total Anthocyanin Content

Standards and falls of flowers at the three stages of development were ground fully and the supernatants were extracted. The absorbance of the supernatants was measured at 530 and 700 nm wavelengths. The following formula was used to calculate the total anthocyanin content: anthocyanin content (mg/g) = ΔA × MW × DF × V/Wt, where ΔA = (A530 − A700) − (A530′ − A700′), MW is the molecular weight of delphinidin glucoside (465.2), DF = 0.062 is the solution dilution factor, V is the volume of the extraction solution, and Wt is the weight of the fresh sample [46].

### 4.2. Identification and Quantitative Analysis of Metabolites

The sample was freeze-dried, ground into powder, and stored at −80 °C until required. Approximately 50 mg of powder was weighted and extracted with 0.5 mL of ethanol/water/hydrochloric acid (500:500:1, *V*/*V*/*V*). Then, the extract was vortexed and centrifuged to collect the supernatants. The residue was re-extracted by repeating the above steps again under the same conditions. The supernatants were collected and filtrated through a membrane filter (0.22 μm, Anpel) before LC-MS/MS analysis. The sample extracts were analyzed using a UPLC-ESI-MS/MS system (UPLC, ExionLC™ AD; MS, Applied Biosystems 6500 Triple Quadrupole). Internal standards of 93 anthocyanins were used for analysis. The absolute volume of the substance in the sample was calculated, and the integral peak area of all detected samples was substituted into the linear equation of the standard curve for calculation.

### 4.3. RNA Extraction, Sequencing, and Annotation

Total RNA was extracted from flowers at the three developmental stages with Trizol reagent (Invitrogen, CA, USA), and its quality was tested. Sequencing was performed on an Illumina HiSeq™ 2500 platform by Novogene (Beijing, China). The raw reads were initially processed through in-house Perl scripts to acquire high-quality reads. Trinity (version 2.0.6) with default settings was used to assemble clean reads into clusters. Among clusters of the same gene, the longest sequence was preserved and designated as a unigene. Unigenes were annotated from databases, including the Kyoto Encyclopedia of Genes and Genomes (KEGG), NCBI non-redundant, and Gene Ontology (GO) databases.

Fragments per kilobase of transcript per million mapped reads (FPKM) was used to estimate gene expression. Threshold |log2FoldChange| > 1 and *p* < 0.05 were used to screen differentially expressed genes (DEGs) and genes enriched on GO and KEGG analyses. 

### 4.4. Weighted Gene Co-Expression Network Analysis (WGCNA)

The R package with default parameters was used to perform WGCNA [47,48], in which an adjacency matrix was constructed and the FPKM values were normalized. The metabolite data were imported into the WGCNA package, and correlations were used to relate the gene modules to various metabolites using default settings.

### 4.5. Integrative Analysis of Transcriptome and Targeted Metabolites

To identify key DEGs related to accumulated metabolites in anthocyanin biosynthesis, we used Pearson correlation coefficients (PCCs) to perform an integrative analysis. If the correlation had a coefficient (*r*) value |PCC| ≥ 0.8 and *p* < 0.05, the DEGs and metabolites were considered to be significantly correlated.

The heat map in Figure 6 represents the overall results of the FPKM cluster analysis, which was performed in R by using unit variance scaling (Z-score) of the Metware Cloud platform, a free online platform for data analysis (https://cloud.metware.cn (accessed on 5 May 2023)).

For phylogenetic analysis, the neighbor-joining method was processed by MEGA 6.0. The tree nodes were evaluated with 1000 bootstrap replicates. The protein sequences of R2R3-MYBs were aligned using the ClustalW program.

### 4.6. Quantitative Real-Time PCR (qRT-PCR) Verification

Quantitative real-time PCR was used to verify the expression levels of 9 genes identified by RNA-seq. RNA extraction and cDNA synthesis were conducted using the Tiangen total RNA extraction kit (Tiangen, Beijing, China) and PrimeScript RT reagent kit with gDNA Eraser (TaKaRa, Beijing, China), respectively. *Tubulin* was used as a control gene for sample normalization. Specific primers were designed according to the coding sequence of candidate genes obtained by RNA-seq (Table S3). qRT-PCR tests were carried out with the Trans Start Top qPCR SuperMix (Transgen, Beijing, China) using the ABI 7500 Fast Real-Time Detection System. The amplification procedure was performed in a 20 μL reaction volume with the PCR program described by Chen [49]. The relative expression levels of the tested genes were calculated using the 2^−ΔΔCt^ method [50]. Each sample was quantified in triplicate.

### 4.7. Statistics Analysis

Data on the anthocyanin content and major anthocyanins among standards and falls at 3 stages were analyzed using one-way analysis of variance (ANOVA, version 9.3; SAS Institute, Cary, NC, USA). Mean comparisons were made using Tukey’s honestly significant difference (*p* ≤ 0.05).

## 5. Conclusions

This study provides important insights into the profiling of anthocyanin accumulation and gene expression patterns in the anthocyanin biosynthetic pathway of the bitone cultivar Clarence during flower development. We believe that the variation in major anthocyanins in the standards and falls causes the unique bitone pattern in this cultivar. Key structural genes exhibit their highest expression at half-color rather than at full bloom, which indicates that these genes function ahead of the full coloration of the flower. The R2R3-MYB transcription factors belonging to the subgroups function-annotated in the anthocyanin biosynthetic pathway *Arabidopsis* were also identified in this study. Functional characterization of key structural genes as well as major TFs will be carried out in future studies.

## Data Availability

The datasets presented in this study can be found in online repositories. The raw reads have been submitted to NCBI SRA (Sequence Read Archive, http://www.ncbi.nlm.nih.gov/sra/ (accessed on 8 February 2023)), under the accession numbers PRJNA940345 and PRJNA940354.

## Supplementary Materials

The following supporting information can be downloaded at: https://www.mdpi.com/article/10.3390/ijms242216462/s1.
