# Supplementary material for: Association Analysis of Transcriptome and Targeted Metabolites Identifies Key Genes Involved in Iris germanica Anthocyanin Biosynthesis
Source: Int J Mol Sci. 2023 Nov 17;24(22):16462. doi: 10.3390/ijms242216462 (PMC10671556; doi:10.3390/ijms242216462)
Supplement: Supplementary file 1 [file ijms-24-16462-s001.zip › ijms-2681865-supplementary.pdf]

Supplementary Material

Table S1. The type and content of anthocyanins of standards and falls in different floral development stages

Code	Compound	Class	Contents (μg/g)					
			S1	F1	S2	F2	S3	F3
1	Cyanidin-3-O-glucoside	Cyanidin	0	0	0	0	0	0.05265
2	Cyanidin-3-O-arabinoside	Cyanidin	0	0	0.004057	0	0	0
3	Cyanidin-3-O-rutinoside	Cyanidin	0	0	0	0.01001	0.02482	0.07361
4	Delphinidin-3-O-arabinoside	Delphinidin	0	0	0	0	0	0.05649
5	Delphinidin-3-O-galactoside	Delphinidin	10.18	8.090	8.815	7.598	1.233	0.7445
6	Delphinidin-3-O-sophoroside	Delphinidin	0.02698	0.02640	0.02880	0.5215	0.07928	1.210
7	Delphinidin-3-O-sambubioside	Delphinidin	0	0	0	0.1017	0.03321	0.2879
8	Delphinidin-3-O-rutinoside	Delphinidin	0	0.3960	0.6218	12.57	22.28	34.34
9	Delphinidin-3-O-(6-O-acetyl)-glucoside	Delphinidin	0.01836	0.02664	0.02266	0	0	0
10	Delphinidin-3-O-glucoside	Delphinidin	0	0.4787	0.3741	12.23	14.41	40.53
11	Malvidin-3-O-sophoroside	Malvidin	0	0	0.01895	0.007346	0.04548	0.01358
12	Malvidin-3,5-O-diglucoside	Malvidin	0	0	0	0	0	0.04093
13	Malvidin-3-O-(6-O-p-coumaroyl)-glucoside	Malvidin	0.2214	0.2721	2.454	1.095	1.802	0.6394
14	Malvidin-3-O-glucoside	Malvidin	0.01585	0.01934	0.03507	0.1427	0.5973	0.8280
15	Malvidin-3-O-(6-O-malonyl-beta-D-glucoside)	Malvidin	0.01697	0.008949	0.04107	0.02654	0.03276	0.02322
16	Malvidin-3-O-5-O-(6-O-coumaroyl)-diglucoside	Malvidin	0.006380	0.006570	0.009163	0.005795	0.01568	0.01032
17	Pelargonidin-3-O-5-O-(6-O-coumaroyl)-diglucoside	Pelargonidin	0.02427	0.02457	0.02202	0.02143	0.002452	0.003095
Code	Compound	Class	Contents (μg/g)					

			S1	F1	S2	F2	S3	F3
18	Pelargonidin-3-O-(6-O-p-coumaroyl)-glucoside	Pelargonidin	2.176	1.798	2.037	1.317	0.9911	0.6681
19	Pelargonidin-3-O-(6-O-malonyl-beta-D-glucoside)	Pelargonidin	0	0	0	0	0.005272	0.007691
20	Pelargonidin-3-O-galactoside	Pelargonidin	0.1116	0.3145	0.1402	0.3398	0	0
21	Pelargonidin-3,5-O-diglucoside	Pelargonidin	0.005654	0.007060	0.007995	0.01045	0	0
22	Peonidin-3-O-(6-O-p-coumaroyl)-glucoside	Peonidin	0.6282	0.4021	0.8343	0.5465	0.8211	0.7402
23	Peonidin-3-O-5-O-(6-O-coumaroyl)-diglucoside	Peonidin	0.02460	0.01620	0.01638	0.009139	0.01184	0.009445
24	Peonidin-3-O-arabinoside	Peonidin	0.001255	0	0.003273	0.003289	0.002417	0.005361
25	Peonidin-3-O-rutinoside	Peonidin	0.01510	0.01435	0.01766	0.01478	0.02430	0.01836
26	Petunidin-3,5-O-diglucoside	Petunidin	0	0	0	0.004964	0.001932	0.009862
27	Petunidin-3-O-5-O-(6-O-coumaroyl)-diglucoside	Petunidin	0	0	0.004619	0	0	0
28	Petunidin-3-O-arabinoside	Petunidin	0	0	0	0	0.01927	0
29	Petunidin-3-O-galactoside	Petunidin	0.1109	0.2134	0.07786	2.188	1.623	7.594
30	Petunidin-3-O-glucoside	Petunidin	0.02113	0.03525	0.01529	0.4309	0.3318	1.513
31	Procyanidin B3	Procyanidin	0	0	0	0	0	0.02140
32	Rutin	flavonoid	0.09472	0.2689	0.3605	0.5468	0.4853	0.7002
33	Naringenin-7-O-glucoside	flavonoid	0.1812	0.4543	0.2361	0.6259	0.2260	0.3932
34	Naringenin	flavonoid	0.03245	0.03344	0.05378	0.05256	0.02998	0.02966
35	Dihydromyricetin	flavonoid	0	1.421	0.1069	2.643	0.5721	1.519
36	Kaempferol-3-O-rutinoside	flavonoid	0.3230	0.7185	0.3339	0.9943	0.2255	1.529

Table S2. The expression levels of candidate genes and transcriptome factors involved in the anthocyanin biosynthetic pathway.

Gene ID	Gene name	Annotation	Expression level (FPKM[z])					
			S1	S2	S3	F1	F2	F3
Cluster-7660.64013	*IgPAL1*	phenylalanine ammonia lyase, partial (*Tulipa fosteriana*)	209.89	255.24	8.24	243.18	277.53	8.61
Cluster-7660.79836	*IgPAL2*	phenylalanine ammonia lyase, partial (*Tulipa fosteriana*)	289.64	410.99	1881.14	391.53	523.52	2002.57
Cluster-7660.88198	*IgPAL3*	phenylalanine ammonia-lyase (*Daucus carota*)	55.38	60.09	462.90	103.95	65.52	501.70
Cluster-7660.57603		phenylalanine ammonia lyase, partial (*Lilium hybrid division I*)	26.22	55.97	521.60	26.99	28.74	610.67
Cluster-7660.60861		phenylalanine ammonia lyase (*Trifolium pratense*)	27.50	32.20	165.60	44.02	41.23	177.04
Cluster-7660.61293		phenylalanine ammonia-lyase (*Iris domestica*)	15.36	34.84	6.32	27.60	38.88	5.20
Cluster-7660.72021		phenylalanine ammonia-lyase 2 (*Narcissus papyraceus*)	82.01	135.17	17.07	114.77	155.74	20.16
Cluster-7660.76938		phenylalanine ammonia lyase, partial (*Tulipa fosteriana*)	142.63	175.07	5.72	171.96	159.08	5.22
Cluster-7660.78047	*IgC4H*	cinnamic acid 4-hydroxylase (*Iris domestica*)	133.12	180.88	39.62	190.20	225.09	35.47
Cluster-7660.78203		cinnamate 4-hydroxylase 2 (*Narcissus papyraceus*)	709.97	897.45	292.17	1165.10	1530.54	365.62
Cluster-7660.124640		putative 4-coumarate--CoA ligase-like protein 8 isoform X2 (Cinnamomum micranthum f. kanehirae)	0.00	0.58	3121.76	0.77	0.93	2603.54
Cluster-7660.117844		4-coumarate--CoA ligase-like 9 (Amborella trichopoda)	0.88	2.18	306.16	1.34	1.97	273.88
Cluster-7660.74316		4-coumarate:CoA ligase-like protein (Albuca bracteata)	95.49	93.35	5.00	110.13	123.31	7.90
Cluster-7660.14422	*Ig4CL*	putative 4-coumarate--CoA ligase-like protein 8 isoform X2 (Cinnamomum micranthum f. kanehirae)	0.17	0.66	630.94	0.05	0.25	490.87

Gene ID	Gene name	Annotation	Expression level (FPKM[z])					
			S1	S2	S3	F1	F2	F3

Cluster-7660.124640		putative 4-coumarate--CoA ligase-like protein 8 isoform X2 (*Cinnamomum micranthum f. kanehirae*)	0.00	0.58	3121.76	0.77	0.93	2603.54
Cluster-7660.117844		4-coumarate--CoA ligase-like 9 (*Amborella trichopoda*)	0.88	2.18	306.16	1.34	1.97	273.88
Cluster-7660.74316		4-coumarate:CoA ligase-like protein (*Albuca bracteata*)	95.49	93.35	5.00	110.13	123.31	7.90
Cluster-7660.106434		4-coumarate--CoA ligase-like 1 (*Elaeis guineensis*)	0.08	0.02	629.21	0.12	0.08	579.64
Cluster-7660.78753		4-coumarate--CoA ligase-like 6 (*Phalaenopsis equestris*)	52.80	62.05	563.57	89.62	80.42	36.76
Cluster-7660.86904		PREDICTED: 4-coumarate--CoA ligase-like 5 isoform X1 (*Nelumbo nucifera*)	1.26	2.24	89.43	1.55	1.23	81.20
Cluster-7660.79841		putative 4-coumarate--CoA ligase-like 8 (*Elaeis guineensis*)	18.05	18.62	120.16	24.38	39.10	141.63
Cluster-7660.79181	*IgCHS*	chalcone synthase (*Iris hollandica*)	1401.91	1781.38	186.72	1706.81	2014.43	347.49
Cluster-7660.83474		chalcone synthase (*Iris hollandica*)	149.43	207.00	222.97	366.88	214.99	219.82
Cluster-7660.79235		chalcone synthase (*Iris hollandica*)	1393.64	1949.27	436.16	1852.25	2357.40	639.96
Cluster-7660.79134		chalcone synthase (*Hibiscus cannabinus*)	78.25	250.13	239.01	360.47	866.73	418.79
Cluster-7660.80852		PREDICTED: chalcone synthase 2-like (*Musa acuminata subsp. malaccensis*)	278.18	419.34	560.07	512.66	664.39	778.47
Cluster-7660.104673	*IgCHI*	ORF124 (*Pinus koraiensis*)	18.82	22.79	11.89	88.28	55.67	33.98
Cluster-7660.77089		chalcone isomerase 2 (*Freesia hybrid cultivar*)	152.84	227.87	67.18	335.75	419.77	62.77
Cluster-7660.72712		BnaC09g30110D (*Brassica napus*)	92.80	95.50	20.40	502.84	141.58	107.23
Cluster-7660.121118		chalcone isomerase 1 (*Freesia hybrid cultivar*)	0.55	0.64	61.01	0.27	0.10	38.51
Cluster-7660.29211	*IgF3H1*	flavanone 3-hydroxylase (*Iris hollandica*)	0.22	1.94	10.14	27.26	142.05	9.19

Gene ID	Gene name	Annotation	Expression level (FPKM^z)					
			S1	S2	S3	F1	F2	F3
Cluster-7660.71026	*IgF3H2*	PREDICTED: flavonol synthase/flavanone 3-hydroxylase-like (*Musa acuminata subsp. malaccensis*)	36.96	123.64	1.77	45.31	252.19	3.69
Cluster-7660.73711		flavonol synthase/flavanone 3-hydroxylase-like (*Asparagus officinalis*)	11.51	19.82	37.17	7.85	11.95	21.24

Cluster-7660.78589		Flavonol synthase/flavanone 3-hydroxylase (*Dendrobium catenatum*)	10.91	20.48	0.02	9.83	21.31	0.17
Cluster-7660.80796		flavonol synthase/flavanone 3-hydroxylase-like (*Asparagus officinalis*)	22.53	32.87	6.59	21.34	28.59	3.42
Cluster-7660.77739	*IgF3'5'H*	flavonoid 3',5'-hydroxylase 1-like (*Asparagus officinalis*)	54.77	64.65	0.32	32.15	29.27	0.23
Cluster-7660.61786		Flavonoid 3',5'-hydroxylase (*Actinidia chinensis var. chinensis*)	13.53	35.32	180.03	25.12	81.42	129.18
Cluster-7660.95316		flavonoid 3'-monooxygenase (*Iris hollandica*)	11.78	121.84	125.25	142.76	778.38	60.01
Cluster-7660.83128	*IgDFR*	dihydroflavonol 4-reductase (*Iris hollandica*)	10.24	19.76	9.36	35.30	110.54	1.75
Cluster-7660.98534	*IgANS*	anthocyanidin synthase (*Iris hollandica*)	0.63	20.67	0.84	42.26	438.21	0.81
Cluster-7660.61071	*IgUFGT1*	anthocyanidin 3-O-glucosyltransferase (*Iris hollandica*)	7.36	66.72	0.12	142.71	533.36	0.04
Cluster-7660.60588	*IgUFGT2*	anthocyanin 5-O-glucosyltransferase (*Iris hollandica*)	3.78	21.48	0.83	308.27	805.16	2.35
Cluster-7660.86486	*IgUFGT3*	UDP-glucose-dependent flavonoid glucosyltransferase UGT703B1 (*Crocus sativus*)	36.27	84.61	287.21	80.53	154.97	285.64
Cluster-7660.79033		UDP-glycosyltransferase 88F3-like (*Phoenix dactylifera*)	126.00	138.61	10.98	114.01	145.05	6.73
Cluster-7660.81509		UDP-glucose-dependent flavonoid glucosyltransferase UGT703B1 (*Crocus sativus*)	153.64	136.20	56.34	124.15	195.44	65.64

Gene ID	Gene name	Annotation	Expression level (FPKM[z])					
			S1	S2	S3	F1	F2	F3
Cluster-7660.73000		UDP-glucose-dependent flavonoid glucosyltransferase UGT703B1 (*Crocus sativus*)	10.98	25.55	95.94	28.27	48.90	88.84
Cluster-7660.76635		UDP-glycosyltransferase 88F3 (Elaeis guineensis)	212.18	193.87	3.54	159.16	155.64	0.59
Cluster-7660.57431		flavonoid glucosyltransferase (plastid) (*Crocus sativus*)	3.34	6.26	272.73	6.07	9.88	220.72
Cluster-7660.82663		Anthocyanin 3'-O-beta-glucosyltransferase (*Dendrobium catenatum*)	76.97	75.49	395.19	77.61	88.81	328.06
Cluster-7660.60309		anthocyanin 5-O-glucosyltransferase (Iris hollandica)	0.61	3.04	0.00	63.84	90.98	0.35
Cluster-7660.59450	*IgAP2/ERF1*	AP2/ERF->AP2/ERF-ERF	37.61	30.22	29.68	38.78	39.03	34.07

Cluster-7660.76315	*IgAP2/ERF2*	AP2/ERF->AP2/ERF-ERF	108.12	82.58	56.43	84.22	102.13	64.86
Cluster-7660.96364	*IgAP2/ERF3*	AP2/ERF->AP2/ERF-ERF	58.80	41.79	31.64	52.54	63.82	31.62
Cluster-7660.72002	*IgbHLH1*	bHLH	72.85	55.20	0.12	32.93	26.26	0.22
Cluster-7660.76887	*IgbHLH2*	bHLH	48.05	21.99	1.29	31.28	17.70	0.70
Cluster-7660.89024	*IgbHLH3*	bHLH	18.08	9.31	0.37	7.92	7.01	0.61
Cluster-7660.76117	*IgbZIP1*	bZIP	48.12	43.86	61.61	48.44	45.81	58.02
Cluster-7660.79673	*IgbZIP2*	bZIP	172.02	99.51	63.08	99.02	86.00	81.77
Cluster-7660.82667	*IgbZIP3*	bZIP	74.29	54.55	9.61	70.52	79.12	7.75
Cluster-7660.84758	*IgbZIP4*	bZIP	102.78	47.13	9.99	72.86	46.83	9.70
Cluster-7660.102699	*IgMYB1*	MYB->MYB	31.68	24.22	155.89	41.86	33.76	161.99
Cluster-7660.66159	*IgMYB2*	MYB->MYB-related	46.12	23.68	0.71	22.86	27.75	0.30
Cluster-7660.73597	*IgMYB3*	MYB->MYB-related	28.92	23.53	0.56	40.72	25.74	0.47
Cluster-7660.74811	*IgMYB4*	MYB->MYB	140.66	52.30	40.74	110.76	70.83	40.14
Cluster-7660.83013	*IgMYB5*	MYB->MYB	68.12	18.49	0.29	41.78	19.43	0.10

[z] FPKM: expected number of Fragments Per Kilobase of transcript sequence per Millions base pairs sequenced.

Table S3. Primers were used in quantitative real-time PCR was used to verify the expression levels of candidate genes.

Gene ID	Primer name	Sequence
Cluster-7660.74312	IgCHI1_F	TCTTCGCTTCACCTTTCC
	IgCHI1_R	TGGTCAACACGGATCAAG
Cluster-7660.84403	IgCHI2_F	AAGCTGGATTGATGGATGG
	IgCHI2_R	GGTTGGCGTTATTGGAATC
Cluster-7660.29211	IgF3H_F	ATCGCAACCTTCCAGAAC
	IgF3H_R	CCTTCTCCGTAACCTTGG
Cluster-7660.95316	IgF35H_F	CCTTGATGACGGCTTGTA
	IgF35H_R	GGCTCTCCTACTGAACCT
Cluster-7660.83128	IgDFR_F	AAGGAATGGTAGAGGAAGAG
	IgDFR_R	TAGTGTGGTGAAGTTGGTTA
Cluster-7660.98534	IgANS_F	GCGTGAGACCTTCCATTG
	IgANS_R	CGGCTTGTGACTGTAGAC
Cluster-7660.57431	IgGT1_F	GGAGTAGCCGTGGAAGTA
	IgGT1_R	CGTCGTCTACACCTTCTTC
Cluster-7660.60588	IgGT2_F	GCCACCAACGATCTCTAC
	IgGT2_R	CTCCTTCTGCTCCTCCTT
Cluster-7660.80885	IgGT3_F	GACCATCATAAGCCAACCT
	IgGT3_R	GAATCTCGCTGCTCTGTT
Reference gene	IgTublin_F	TGGTTAGAAAGGAGGCAGAGAATTG
	IgTublin_R	CAGTTTCCCATACCAGATCCAGTC
